# Comparison of Phenolic Content and Antioxidant Activity for Fermented and Unfermented Rooibos Samples Extracted with Water and Methanol

**DOI:** 10.3390/plants11010016

**Published:** 2021-12-22

**Authors:** Eslam A. Hussein, Christopher Thron, Mehrdad Ghaziasgar, Mattia Vaccari, Jeanine L. Marnewick, Ahmed A. Hussein

**Affiliations:** 1 Department of Computer Science, University of the Western Cape, Bellville, Cape Town 7535, South Africa; ehussein@uwc.ac.za (E.A.H.); mghaziasgar@uwc.ac.za (M.G.); 2 Department of Science and Mathematics, University-Central Texas, Killeen, TX 76549, USA; thron@tamuct.edu; 3 Department of Physics and Astronomy, University of the Western Cape, Cape Town 7535, South Africa; mvaccari@uwc.ac.za; 4 Applied Microbial and Health Biotechnology Institute, Cape Peninsula University of Technology, Symphony Road, Bellville, Cape Town 7535, South Africa; marnewickj@cput.ac.za; 5 Department of Chemistry, Cape Peninsula University of Technology, Symphony Road, Bellville, Cape Town 7535, South Africa

**Keywords:** rooibos tea, *Aspalathus linearis*, phenolics, antioxidant, classification, small data, machine learning, water extract, methanol extract, baseline, uncertainty quantification

## Abstract

Rooibos is brewed from the medicinal plant Aspalathus linearis. It has a well-established wide spectrum of bio-activity properties, which in part may be attributed to the phenolic antioxidant power. The antioxidant capacity (AOC) of rooibos is related to its total phenolic content (TPC). The relation between TPC and AOC of randomly selected 51 fermented (FR) and 47 unfermented (UFR) rooibos samples was studied after extraction using water and methanol separately. The resulted extracts were assessed using two antioxidant assays, trolox equivalent antioxidant capacity (TEAC) and ferric reducing antioxidant power (FRAP). The results were analyzed using both simple statistical methods and machine learning. The analysis showed different trends of TPC and AOC correlations of FR and UFR samples, depending on the solvent used for extraction. The results of the water extracts showed similar TPC and higher AOC of FR than UFR samples, while the methanolic extracted samples showed higher TPC and AOC of UFR than FR. As a result, the methanolic extracts showed better agreement between TPC and AOC than water extracts. Possible explanations are given for these observed results. Although, the current literature demonstrates direct correlations of the TPC and AOC of rooibos water extracts. This study showed deviation and highlighted the importance of solvent selection and analysis methodology as an important factor in determining the TPC/AOC correlation and subsequently the expectation of the actual health benefits of rooibos herbal tea. In particular, unfermented and fermented samples can be accurately identified on the basis of a combination of assays (any two of TPC, FRAP and TEAC), especially if methanol is the solvent used. Machine learning analysis of assay data provides nearly identical results with classical statistical analytical methods. This is the first report on machine learning analysis and comparison of the TPC and AOC of rooibos herbal tea extracted with methanol and water, and highlights the importance of using methanol as a solvent to evaluate its AOC.

## 1. Introduction

Rooibos is an indigenous South African plant that grows only in the Cederberg region approximately 300 km north of Cape Town. For centuries, natives of the region have processed its leaves to prepare herbal tea [1]. Recently, South Africa’s rooibos herbal tea has become the first African food to receive approval for registration under the status of international protection from the European Union [2]. This inclusion will preserve the long-standing association between rooibos and South Africa. Compared to other teas such as black tea, rooibos has several distinctive biochemical properties with potential health benefits. Those benefits are attributed to the antioxidant properties of polyphenolic compounds [3].

Given the importance of rooibos as a herbal tea, and due to its health benefits as an antioxidants agent, determining antioxidant activity is of special interest. In particular, the correlations between the antioxidant properties and polyphenolic content in UFR and FR samples have significant practical and commercial implications [4]. To date, there is no clear biomarker for FR and UFR to be used as a standard that can reflect the health quality, rather, total phenolic content (TPC) has been used as a first line in evaluation of the quality of tea products [5], accompanied by in vitro chemical antioxidant assays such as DPPH [5,6,7,8,9,10,11], ORAC [5,11,12,13], FRAP [6,10,13,14], ABTS [12,15,16], TEAC [14,17], and β-carotene bleaching [7,8].

The literature also indicates a direct correlation between the TPC and antioxidant capacity of rooibos as measured by one or a combination of some assays mentioned above. However, the reported correlations are not consistent due to the fluctuation of phenolic constituents among the FR and UFR final products [6,7,15,16,18,19,20] and contradictions exist among the reported data. Reference [6] showed direct correlation between DPPH and FRAP activities with TPC, while [7] measured the antioxidant activities using DPPH and β-carotene bleaching methods, and showed the FR and UFR samples have relatively equal amounts of TPC, and UFR showed slightly higher DPPH inhibition activity than FR, while FR showed higher activity than the UFR samples using the β-carotene test. In another study, one of the rich phenolic containing fractions (EtOAc from UFR) demonstrated low antioxidant activity [21]. These contradictory results provide motivation for the current study, which quantifies the relation between TPC and two antioxidant assays using different solvents for fermented and unfermented rooibos.

Plant phenolic constituents are the major natural constituents that contribute to the antioxidant activity [10,11,14,22,23,24,25], and most of these compounds are hydrophillic in nature (glycosylated) [10,14,25]. Solvent extraction is the first step in analysis of TPC of plant materials, which depends on the solvent polarity used, and is essential for accurate quantification of Antioxidant Capacity (AOC) [10,26]. In addition to the solvent, the physical nature as well as chemical structure complexity are important factors, among others affect the efficiency of the extraction methods [13]. For determination of AOC, methanol and water are the most popular and efficient solvents in extraction of phenolic components [13,22,23], in addition to different aqueous alcohol or acetone mixtures [10,13,23].

There are standard statistical methods for developing classification models based on sample data. However, in the past few decades, machine learning (ML) has shown improvements over statistical categorization methods in many applications. Recently, several authors in the plant chemistry field have employed ML techniques to discover associations between chemical properties and food characteristics [4,27,28,29,30,31,32,33,34,35]. At the same time, some authors have cast doubt on the efficacy of ML when relatively small samples are used [36,37,38] (e.g., less than a few hundred), which is typically the case in food chemistry applications. For this reason, in addition to conventional methods, we also apply ML to the problem of classifying samples into FR and UFR based on the phenolic conetent and antioxidant activities, and do a rigorous comparison of the accuracy of these methods compared to standard statistical techniques.

In this research, we are interested in the differences in TPC and AOC between FR and UFR samples, as well as the effectiveness of different solvents (methanol and water) for extracting compounds with antioxidant properties. Thus, two antioxidant assays were performed on UFR and FR tea samples extracted with methanol and with water. Both classical statistical methods and ML were applied, and the results were compared in the analysis and classification of FR and UFR samples based on the phenolic content and antioxidant properties measured using the two solvents.

## 2. Materials and Methods

### 2.1. Chemicals

Trolox (6-hydroxy-2,5,7,8-tetramethylchroman-2-carboxylic acid, purchased from Sigma Aldrich, Cape Town, South Africa) was used as the antioxidant standard. ABTS•+ [2,2′-azinobis-(3-ethylbenzothiazoline-6-sulfonic acid) diammonium salt], gallic acid, and Folin Ciocalteu’s phenol reagent were obtained from Sigma-Aldrich (Cape Town, South Africa). Methanol was purchased from local market (Kimix, Cape Town, South Africa). Standards were dissolved in methanol (1 mg/mL) and stored at −20 °C in a freezer.

### 2.2. Extraction Procedures

Ninety-eight randomly selected FR (51 samples) and UFR (47 samples) were kindly donated by Rooibos LTD-BPK (Clanwilliam, South Africa) during March 2020. The fermentation process of the green leaves/stems was performed at ambient temperature of 35–38 °C for 6–8 h, under a relative humidity of 65%. UFR samples were prepared by fast drying the green leaves/stems at 100 °C for 20 min. All samples were extracted using both water and methanol, as described in the following paragraphs.

Water extracts were obtained by pouring 200 mL of freshly boiled (90 °C) distilled water on 10 g of plant materials and steeping for 10 min. The infusions were left to cool to room temperature, then decanted and centrifuged at 3000 rpm for 30 min, and lyophilised (Virtis Genesis 25EL, Stone Ridge, NY, USA). The resulting powders were kept at room temperature in dry conditions until further use. Methanolic extraction was performed by addition of 200 mL of methanol to 10 g of plant materials, heated for 2.0 h in a water bath at 60 °C and filtered. The filtrate was then concentrated using rotatory evaporator (Buchi, Postfach, Switzerland) and the residue kept at −5 °C until further use.

### 2.3. Chemical Assay Procedures

#### 2.3.1. Total Polyphenols

The evaluation of the total amount of phenolics was done using the method of Singleton and colleagues with slight modifications [39]. Following prescribed protocols, plates containing the extracts were read at 593 nm and the results were expressed as gallic acid equivalents (GAE).

#### 2.3.2. Trolox Equivalent Absorbance Capacity (TEAC) Assay

The TEAC assay was evaluated following the method of Re et al. in [40]. The working solution containing 88 μL of K_2_S_2_O_8_ (140 mM) and 5 mL ABTS (7 mM) was kept for at least 16 h in the dark at 25 °C. The working solution was then diluted with ethanol until the absorbance read approximately 2.0 (±0.1). The extract, purified compounds, or standard (25 μL) was mixed with 300 μL working solution and allowed to incubate in the dark for 30 min at room temperature. Trolox was used as the standard using a concentration range between 0 and 500 μM. The absorbance was read at a wavelength of 734 nm using a multiplate reader (SpectraMax i3X, San Jose, CA, USA).

#### 2.3.3. Ferric-Ion Reducing Antioxidant Power (FRAP) Assay

The FRAP assay was assessed according to the method of Benzie and Strain [41]. FRAP reagent containing the mixture of acetate buffer (300 mM, pH 3.6), tripyridyl triazine (TPTZ) (10 mM in 40 mM HCl), and 20 mM FeCl_3_·6H_2_O in ratio 10:1:1 (*v*/*v*/*v*) was used. Extract, or standard (10 μL) was added to 300 μL FRAP reagent, incubated for 30 min in the dark at room temperature. The reacting mixture was read at a wavelength of 593 nm in a multiplate reader. Ascorbic acid was used as standard at varying concentrations of 0 to 1000 μM. The result was presented as a mean of independent triplicate experiments and expressed as μM ascorbic acid equivalents per milligram dry weight (μM AAE/g) of the test samples.

### 2.4. Database Creation

TPC, TEAC, and FRAP measurements were repeated three times for each of the 98 samples. The Half-maximal inhibitory concentration (IC_50_) was calculated using GraphPad Prism 5 version 5.01 (Graph pad software, Inc., La Jolla, CA, USA) statistical software. The data presented are means and standard deviations obtained from 96-well-plate readers for all in vitro experiments.

### 2.5. Statistical Analysis

To analyze distributions of TP and antioxidant activities for FR and UFR samples, histograms were used. To investigate relationships between chemical characteristics, bivariate scatter plots were created for FR and UFR separately, and 95% confidence ellipses were calculated and plotted. The corresponding measurements for two solvents were compared, and different measurements for each solvent were compared pairwise.

### 2.6. Statistical and Machine Learning Classifiers

Statistical and ML methods for were used to classify samples into FR and UFR, based on measured activity data. In order to understand the relative importance of different features in distinguishing between FR and UFR, we developed binary classifications based on the following feature sets:

- TP only;
- TEAC only;
- FRAP only;
- TP, TEAC;
- TP, FRAP;
- TEAC, FRAP;
- TP, TEAC, FRAP.

These seven feature sets were used for each solvent separately and for both combined, making a total of 7×3=21 different classifiers.

ML always involves testing and training phases. Testing typically involves some form of cross-validation, to avoid over-fitting. To our knowledge, there are no applicable systematic studies on the relative effectiveness of different cross validation methods. For this reason, several alternative cross-validation configurations were tried, and jackknife estimation was used to determine variability of each alternative. Based on this investigation, a 1/2-1/2 split of data into training and testing sets was used, with 3-fold cross-validation on the training set. Training and testing sets were created using random stratified sampling.

Both statistical and ML classifiers were investigated, and their performances compared. The following subsections describe the classifiers used and the methodology used to compare performance.

#### 2.6.1. Statistical Baseline Method

First, a simple statistical classifier was used, in order to have a benchmark against which ML methods could be evaluated. For this purpose, we used maximum likelihood estimation based on Gaussian fit of the data. Specifically, we first computed means and covariances of feature sets separately for FR and UFR, based on testing data. Then, to classify a data point, we computed *Z*-scores for the two separate distributions, and assigned the classification associated with the smaller absolute multivariate *Z*-score. Note that this is equivalent to finding the minimum Mahalanobis distance between the two distributions [42].

#### 2.6.2. Machine Learning Classifiers

A total of four ML tools are used for the binary classification: (a) Logistic regression (LR), (b) support vector machine (SVM), (c) random forest (RF), *k*-nearest neighbor (Knns). These methods are state-of-the-art for ML with small datasets [38,43,44,45].

We did not consider neural network methods, which require larger amounts of data [46,47,48,49]. For the optimization, we used grid search, which was applied on every feature set and data split described above, implemented in scikit-learn [50] and Python programming language.

#### 2.6.3. Comparison of Classifiers’ Performances

In this research, we compared the classification accuracies of all ML methods for all feature sets with the corresponding baselines, which are computed as follows [51].

(1) $$Accuracy=\frac{TP+TN}{TP+TN+FP+FN}$$

where TP is the true positive, TN is the true negative, FP is the false positive, and FN is the false negative.

Since the number of instances in each class was nearly equal, recall and precision values do not give additional insight, and were not computed. To make an effective comparison, error bars for the classification accuracy differences were also calculated. If zero difference lay outside the error bars of the estimated difference, then we concluded that the difference between estimators was statistically significant. Otherwise, we failed to reject the null hypothesis of no difference between estimators.

The accuracy of the different classifiers were evaluated using the testing set. In order to obtain error bars, jackknife with leave-out-one was implemented. Each of the leave-out-one instances was optimized separately. This required intensive computation: since there were 98 reduced data sets of 97 points (each obtained by leaving out one data point), it follows that 98 parameter optimizations were performed for each ML method for each feature set. Altogether, this amounted to 98(datasets)×21(featuresets)×4(MLmethods)=8232 separate optimizations.

## 3. Results

### 3.1. Overview

The randomly selected 51 FR and 47 UFR rooibos samples were extracted with water and with methanol, and the extracts were evaluated for their total phenolic content, as well as antioxidant capacity using the TEAC and FRAP assays.

### 3.2. Determination of Total Phenolic in Fermented and Unfermented Samples

**Figure 1:**
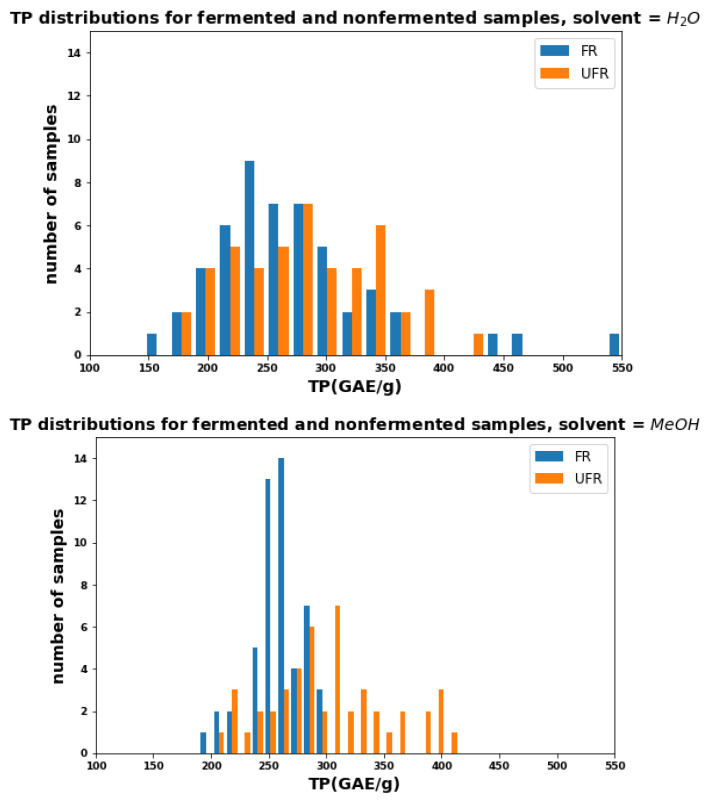
Distributions of total phenolics for FR and UFR using water (**top**) and methanol (**bottom**) as solvents.

Figure 1 shows the relative distribution of the TPC among the water and methanolic extracts of fermented (FR) and unfermented (UFR) samples. The samples extracted with water showed similar phenolic distributions with nearly identical means (279 for FR vs. 282 GAE/g for UFR), while the samples extracted with methanol showed generally higher phenolic content in the UFR samples, and the mean for FR is 16% lower (257 for FR vs. 303 GAE/g for UFR).

**Figure 2:**
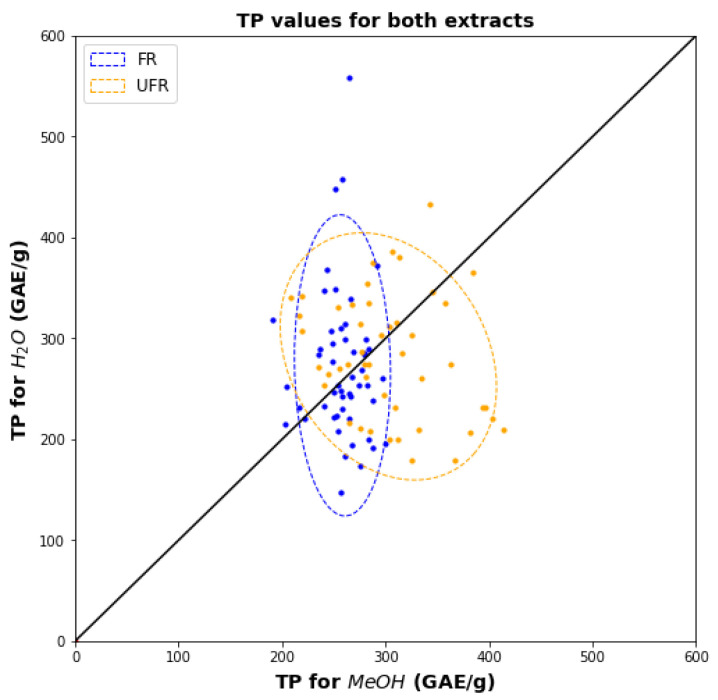
Scatter diagram showing FR and UFR TPCs for water versus methanol.

Figure 2 is a scatter diagram showing the joint distributions of water and methanol TPCs for FR and UFR separately. The 95% confidence ellipses for the two distributions are also shown. For both FR and UFR samples the two TPCs show little to no association, showing that methanol and water do not extract the same phenolics. Compared to UFR, the FR samples show a broader range of water TPCs, but a much narrower range of methanol values. Most FR data points also lie inside the UFR confidence ellipse, which indicates that the two TP assays fail to distinguish FR samples from UFR.

### 3.3. Determination of TEAC in Fermented and Unfermented Samples

**Figure 3:**
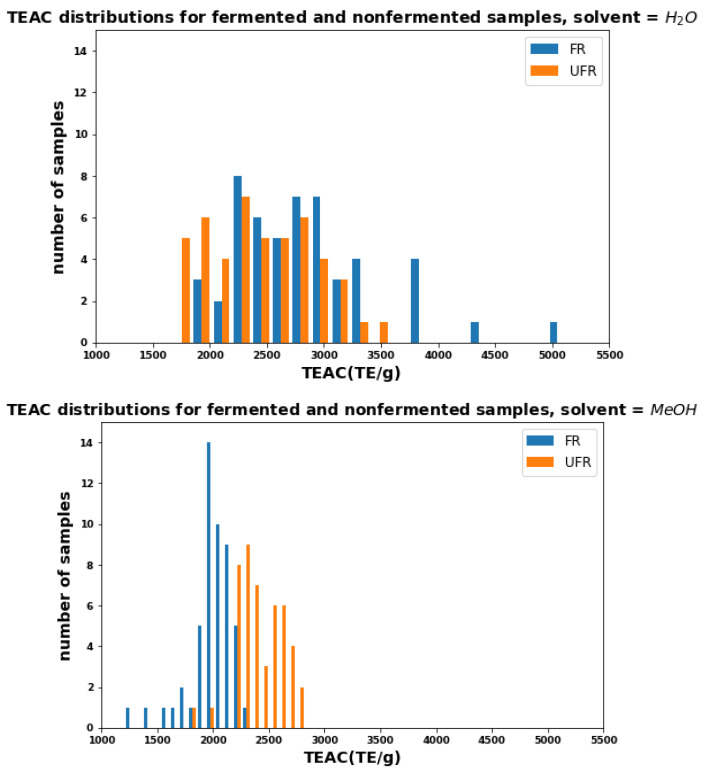
Distributions of TEAC for FR and UFR using water (**top**) and methanol (**bottom**) as solvents.

Figure 3 shows histograms for AOC in water (top) and methanol (bottom) as measured by TEAC. These graphs resemble the two TPC histograms in Figure 1. As with TPC, the distributions for FR and UFR, TEACs with water are similar (except for a few FR outliers), while in methanol, the FR distribution is visibly narrower and lower than UFR (the mean is reduced by 21%).

**Figure 4:**
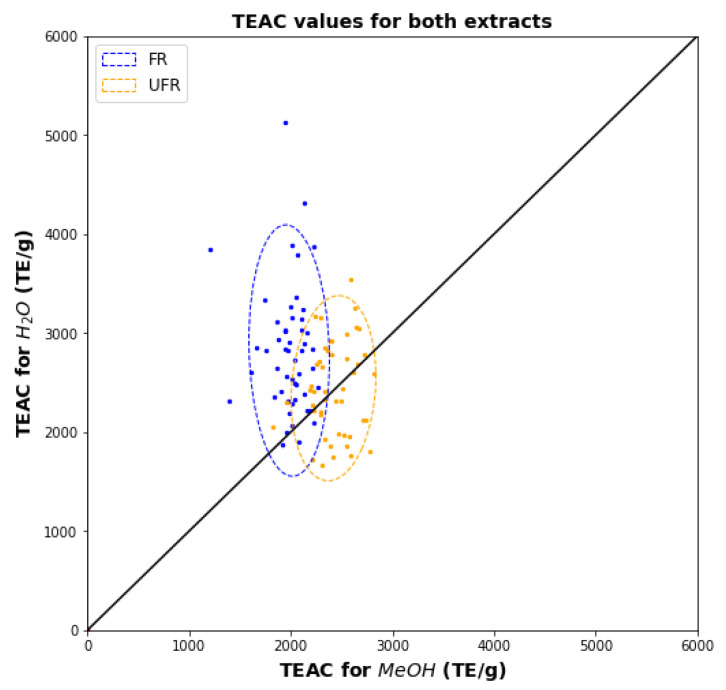
Scatter diagram showing FR and UFR TEACs for water versus methanol.

Figure 4 shows a scatter plot for the two solvents’ TEAC values for FR and UFR samples. As observed for TPC in Figure 2, the ranges of TEAC values in methanol are much narrower than water, and there is no association between the TEAC values for the two solvents. The overlap between FR and UFR confidence ellipses is reduced compared to TPC (compare Figure 2), which implies that TEAC assays can better characterize the difference between FR and UFR than TPC assays.

### 3.4. Determination of FRAP in Fermented and Unfermented Samples

**Figure 5:**
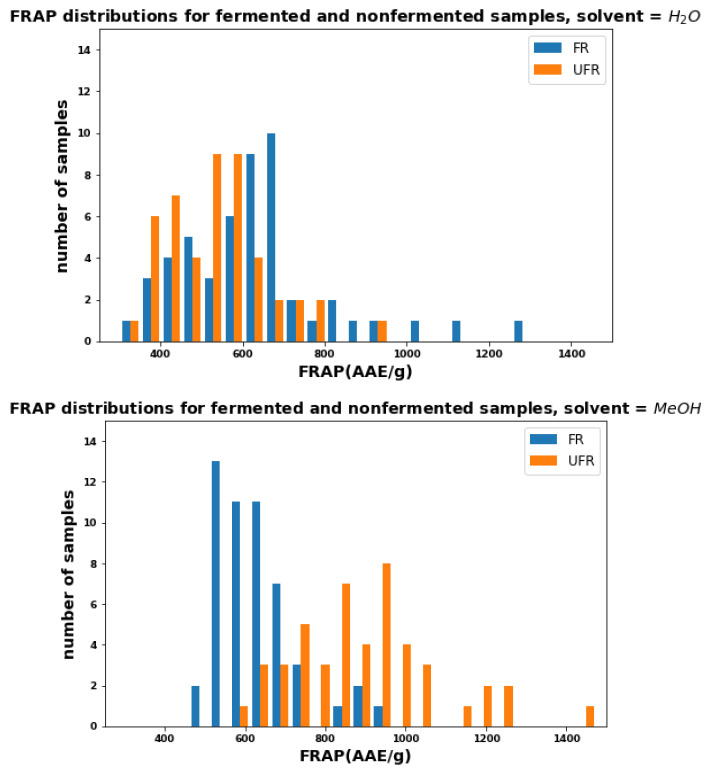
Distributions of FRAP for FR and UFR using water (**top**) and methanol (**bottom**) as solvents.

Figure 5 shows the AOC as measured by the FRAP assay for FR and UFR samples extracted with water (top) and methanol (bottom). As in Figure 3, FR and UFR samples extracted with water showed relatively equal antioxidant activities. The difference between UFR and FR methanol-extracted samples is even more extreme than those observed with TPC and TEAC, and some UFR samples have more than twice FRAP values than FR sample.

**Figure 6:**
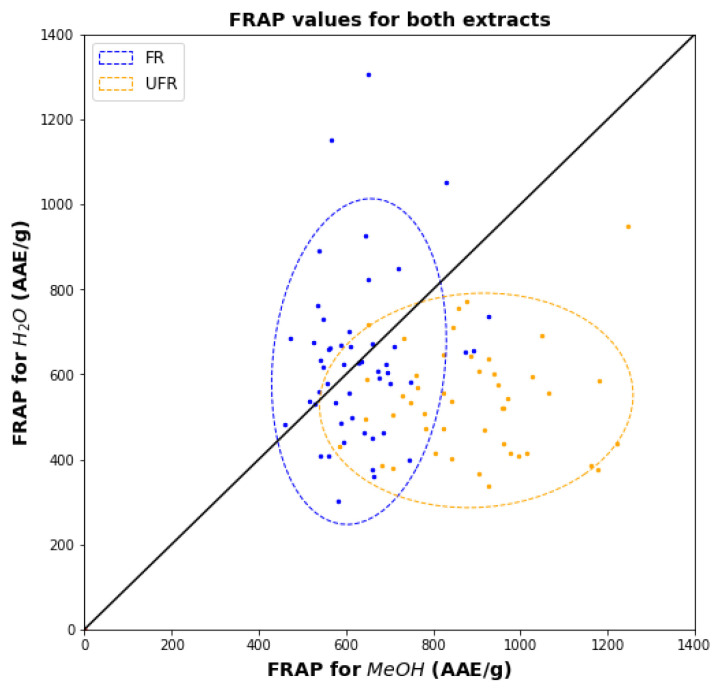
Scatter diagram showing FR and UFR FRAPs for water versus methanol.

FRAP for FR has similar characteristics as TPC and TEAC (Figure 6). However, the FRAP for UFR shows a much wider range of values for methanol and a narrower range for water. The overlap between FR and UFR regions is small.

### 3.5. Assay Joint Distributions and Correlations

In this section, we investigate pairwise statistical relationships among the distributions generated by the three different assays (TPC, TEAC, FRAP), for FR and UFR for each solvent separately.

**Figure 7:**
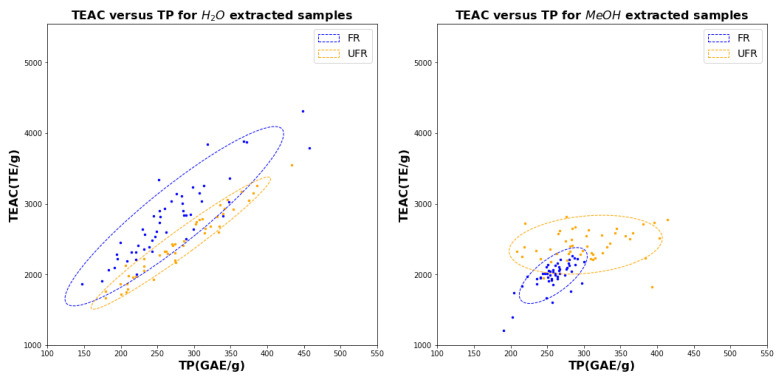
Scatter diagram for TP versus TEAC using water (left) and methanol for 51 FR samples (blue) and 47 UFR samples (orange).

**Figure 8:**
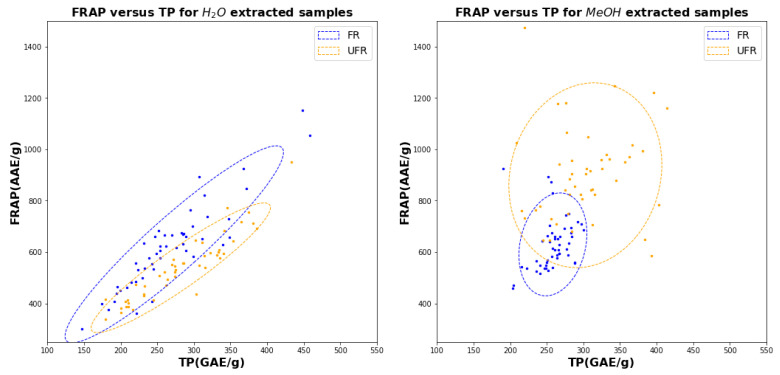
Scatter diagram for TP versus FRAP using water (left) and methanol for 51 FR samples (blue) and 47 UFR samples (orange).

**Figure 9:**
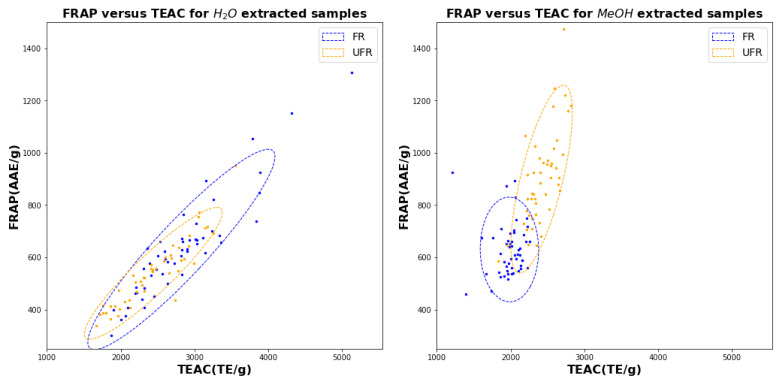
Scatter diagram for TEAC versus FRAP using water (left) and methanol for 51 FR samples (blue) and 47 UFR samples (orange).

The scatter diagrams in Figure 7, Figure 8 and Figure 9 make pairwise comparisons between the the three assays (TPC, TEAC, and FRAP) for water (left) and methanol (right). Since each figure includes FR and UFR comparisons, these figures represent altogether 12 bivariate comparisons. The squared correlation coefficients ($R^{2}$ values) with uncertainties for all 12 comparisons are listed in Table 1.

It is immediately clear from Table 1 that all water assays are highly correlated, with all correlations exceeding 0.9: these correlations are also evident in the linear shapes of the scatter clouds in Figure 7, Figure 8 and Figure 9. On the other hand, for methanol, the only significant correlations are between TEAC and FRAP for UFR, which both show correlations close to 0.7.

The figures also show that water is much less effective in distinguishing FR from UFR, because the FR and UFR confidence ellipses in the figure for water on the left have much less overlap than those in the corresponding figure for methanol on the right. In particular, Figure 9 (*left*) shows that TEAC and FRAP in water give basically the same information, which is unaffected by fermentation.

We may note additionally that the confidence ellipses for FR in water are consistently larger than the confidence ellipses for UFR, indicating that FR assay values have more variability than UFR when water is used as a solvent. However, when methanol is used, the FR confidence ellipses are smaller than the UFR ellipses, indicated reduced variability for FR samples.

### 3.6. Statistical and Machine Learning Classifications

In recent years, ML has become an increasingly popular alternative to classical statistics as a method for classifying samples based on data. This popularity has extended to the field of food chemistry references. For this reason, we employ both classic statistical and ML classifiers, as described in Section 2.6. In the next subsection, we present statistical classifiers, and the subsequent subsection compares a statistical baseline classifier with ML classifiers.

#### Statistical Classifications of FR versus UFR

**Figure 10:**
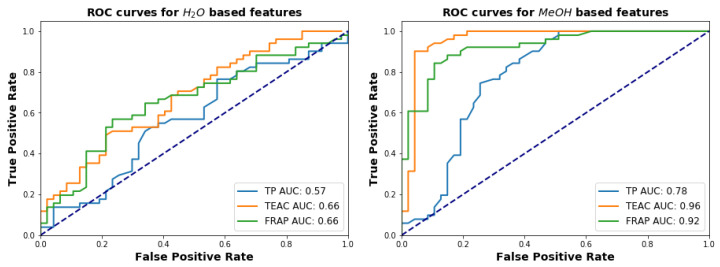
ROC curves for the three assays, for water (**left**) and methanol (**right**) extracts.

To quantify how individual assays fare in distinguishing FR from UFR, we compute six receiver operator characteristic (ROC) curves [52] based on thresholds imposed individually on the six different assay-solvent combinations. The three curves for water assays and for methanol assays are shown in Figure 10 left and right, respectively. The methanol assays are clearly superior for the purposes of distinguishing FR from UFR, with area under curve (AUC) values ranging from 0.78 to 0.96 compared to a range of 0.57–0.66 for water assays.

In order to show how multiple assays taken together may improve classification, we determined the accuracy of statistical classifiers (as defined by Equation (1)) based on multiple features. These classifiers are based on Mahalanobis distance, as described in Section 2.6. We take these classifiers as our baselines, which will be compared in the next subsection with ML classifiers.

**Figure 11:**
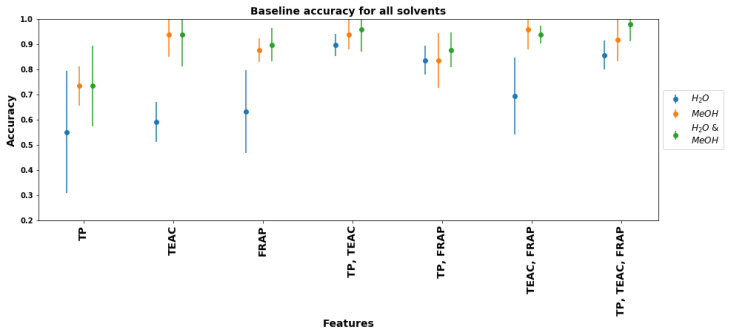
Accuracies of baseline classifiers for FR and UFR based on seven different feature sets using extraction data from water only, methanol only, and both water and methanol. Error bars show two standard deviations.

Figure 11 shows the accuracy of baseline classifiers for distinguishing FR from UFR based on different combinations of assays. As a general rule, methanol-based assays give more accurate classifications than water-based: this is consistent with the ROC curves shown in Figure 10. Using both methanol and water-based assays together gives minimal improvements over methanol only. Additionally, all methanol-based classifiers that employ TEAC perform well; those that employ FRAP are slightly worse. Of the water-based classifiers, those that use TP and least one other factor (either TEAC or FRAP) achieve good accuracy (∼90% or above), but single-factor water-based classifiers are all poor performers with ∼55–65% accuracy.

### 3.7. Comparison of Statistical and Machine Learning Classification

In order to make apples-to-apples comparisons of different classification methods, we compare classifiers based on the same features using different ML classifiers and compare the accuracy with the baseline statistical estimates described in the previous subsection. The method for estimating relative classifier accuracies and error bars is described in Section 2.6.

**Figure 12:**
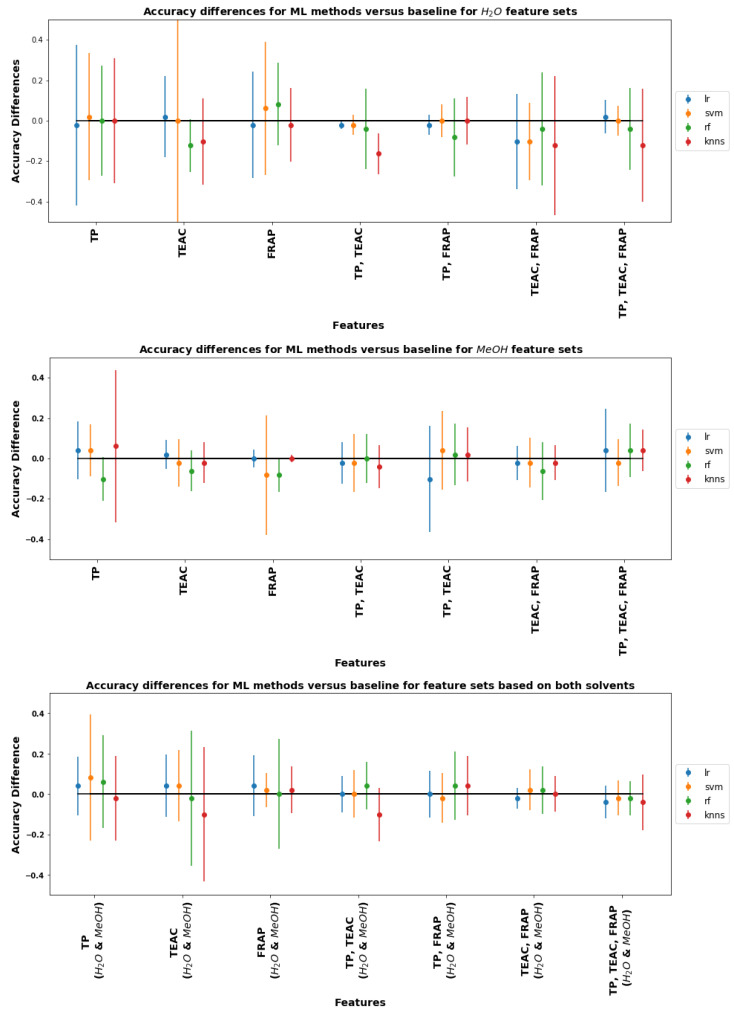
Final results of accuracy differences across all ML methods for classifiers based on water only (**top figure**), methanol only (**middle figure**) and both water and methanol (**bottom figure**).

Figure 12 shows the accuracy differences between the baseline statistical estimator and ML-based estimators for all different solvent-assay combinations. The figures show that none of the ML methods give significant improvements in accuracy over the baseline. In fact, in many cases, the estimated accuracy is lower than the baseline accuracy for the simple statistical estimator (although the differences are not statistically significant).

## 4. Discussion

Rooibos herbal tea has a wide spectrum of pharmacological activities that are related to its antioxidant capacity. Most prior publications attribute the AOC to the phenolic content. Although FR and UFR rooibos differ in their chemistry, both have proven AOC. The variation of phenolic content due to environmental conditions and processing is well documented [9,11,12,15,18], which decreases the possibility of finding a single bio-marker that is responsible for its health benefits [15]. The determination of TPC as a direct reflection of the AOC has gained wide acceptance in industrial practice as well as from academic researchers [6,8,15,53].

### 4.1. Water Extractions

Table 2 shows that in the case of TPC measured using water extraction, UFR and FR show similar averages. These results are supported by reference [9], which reported TPCs of 40.99 GAE g/100g extract for UFR and 34.95 GAE g/100 g extract for FR. Reference [53] also found nearly equal averages TPC of UFR and FR extracted with water. However, [12] used different extraction times and water as a solvent and showed 23.7–56.8% TPC decrease in FR samples; while reference [15] extracted different samples using water, and obtained TPC values for UFR nearly twice as large as for FR (8.12 versus 4.54 GAE/100 g dry wt). The wide variation in these results can be attributed to many factors as mentioned before, and most importantly, the environmental growing conditions and processing including extraction conditions. For example, reference [18] indicates the presence of clear differences in TPC (and individual phenolic compounds) among wild populations of rooibos collected from different natural habitats. The comparison of the TPC for black and green tea showed almost equal values (17.0 green/16.5 black g/kg) [24].

The average values of both TEAC and FRAP for UFR are lower than corresponding values for FR (Table 2). These results differ from previously reported data, where FRAP values of FR samples was higher than UFR, which is unexpected because the FRAP assay eliminates the contribution of protein and amino acids to the antioxidant capacity of phenolic compounds. The UFR contains high concentration of aspalathin which considered to be a potent antioxidant agent compared to other phenolic constituents in rooibos [8]. However, such variations can be supported by the fact that the fermentation process, while decreasing the phenolics content (specially dihydrochalcones), also forms more stable flavonoids and brings all the phenolics to the surface where they are more readily extracted than in UFR. The other explanation could be that the non-phenolics content (such as carbohydrates, proteins, amino acids, chlorophyll degradation products, inorganic salts, etc.) after fermentation becomes more freely accessible and forms real natural complexes with the phenolic compounds. These complexes are expected to have synergetic effects that contribute to the final AOC. This assumption is supported by the reduced AOC in FR compared to UFR when methanol was used as solvent, because of its limited polarity, which avoids the extraction of non-phenolic polar constituents.

### 4.2. Methanol Extracts

Contrary to water, methanol extraction showed clear discrimination between the TPC of UFR and FR. We could find no previous comparison in the literature between methanolic extracts for UFR and FR. However, Ref. [54] indicated relative high phenolic content of aqueous extracts (25% *w*/*w*) compared to ethanolic extracts (23% *w*/*w*). The low TPC in FR samples are in agreement with the decrease of phenolic compounds during the fermentation process, and these results have been supported by many reports [8,17,53,55,56,57] and showed that dihydrochalcone derivatives (mainly aspalathin) decrease to less than 10% of their initial concentrations. The data also showed higher TPC of UFR samples extracted with methanol than the corresponding samples extracted with water, but lower for FR.

The TEAC of UFR samples were higher than FR. The values are in agreement| with the TPCs of both UFR and FR. Similarly, FRAP showed a higher average for UFR than FR.

### 4.3. Comparison of Water and Methanol Extracts

The polarity of a given solvent affects its ability to dissolve a selected group of antioxidant compounds, thus influencing the antioxidant activity estimation. Compared to water, methanol increases the extraction of lipophilic antioxidant compounds and it may give a better indication of the effects of antioxidants on the body.

The average TPCs of UFR samples extracted with water is lower than UFR samples extracted with methanol, while for FR sample extracted with water showed higher average than samples extracted with methanol. These observations are in agreement with the effect of fermentation on the dihydrochalcones particularly with samples extracted with methanol.

Comparing the TEAC average values UFR samples extracted with water against methanol, showed relatively equal values. On the other hand, the FR samples showed higher average values for water than methanol. While the FRAP average value for UFR/water is lower than UFR/methanol, in the case of FR samples, the average value for FR/water is relatively equal to FR/methanol.

The relatively equal values of TPC, TEAC and FRAP between FR and UFR extracted with water may reflect the fact that water extracts other compounds in addition to phenolics, which also contribute to antioxidant activity. As a result, TEAC and FRAP measurements may overestimate phenolic activity. On the other hand, the type of the plant materials (either fermented or unfermented) also plays an important role in determining the final observed values; however, this is only observed when methanol was used as solvent.

The water extract showed superiority of extraction for TPC (followed by methanol) from black tea and mate tea when compared with acetone, and ethanol. Additionally, the antioxidant measured using DPPH showed the same trends for both teas [58]. Bhebhe et al. indicated that the free radical scavenging activity (FRSA) was not necessarily in the same order as TPC, and the high TPC does not always mean high FRSA and vice versa [21].

### 4.4. Classification Comparisons

The classification of samples into FR and UFR based on water-extracted and methanol extracted samples highlights the considerable differences between the two solvents. The ROC curves in Figure 10 show that, compared to water, each of the three assays with methanol are more able to detect the chemical differences produced by fermentation. As mentioned above, this can be attributed to the fact that water extracts a wider variety of antioxidant compounds, and not just phenolics.

From the baseline classification accuracy results in Figure 11, we may draw several conclusions. First, TPC and AOC of methanol extracts are more effective in distinguishing FR from UFR. Second, using multiple assays as features can improve classification accuracy, especially with assays that use water as solvent. Finally, using TPC and AOC assays of both water and methanol extracts as features does not improve over using methanol-based assays only.

The conclusions in the preceding paragraph may be interpreted in light of the scatter diagrams in Figure 7, Figure 8 and Figure 9. For example, Figure 7 shows that clusters representing FR and UFR samples in methanol are more clearly distinguished than the corresponding clusters for FR and UFR samples in water. Note that both TEAC and TPC coordinates are needed to clearly show the separation, indicating that both assays are required to make an accurate determination. The accuracy difference graphs in Figure 12 show that none of the ML methods tried gave significant improvements in classification accuracy. We may also understand this from the scatter diagrams in Figure 7, Figure 8 and Figure 9. ML is good at classification in cases where there are complicated nonlinear relationships between different features: but the scatter diagrams show on the contrary that sample joint distributions have a simple, unimodal, Gaussian-like shape. Classical statistics is theoretically optimal for Gaussian distributions, so in such cases it is theoretically impossible to improve on the results of classical statistics.

Our results about the non-improvements due to ML support a skeptical outlook towards other similar applications of ML in food chemistry. Some references that apply ML to food chemistry do not compare their results to a statistical baseline, so it is impossible to tell whether ML actually worked better than statistics [27,32,59,60,61]. Furthermore, ML is well-known for being prone to misapplication and misinterpretation [36,37,38]. For example, the good results from ML reported in [4,31] are in fact due to data leakage: different measurements from the same sample were included in both training and testing sets.

## 5. Conclusions

The given data showed that no real reflection or correlations can be made between different types of rooibos when water was used as a solvent. The expected TPC decrease due to fermentation was not observed and the AOC of FR was higher than the UFR. This raises a question as to which are the real compounds that contribute to the final AOC in the FR. On the other hand, methanol showed selective extraction to phenolic compounds as reflected from the average TPC of the extracted UFR and FR samples. This indicates that methanol is preferable to water as a solvent in characterizing the properties of rooibos samples that relate to health benefits. The study also indicates that any two of the TPC, FRAP and TEAC assays using methanol are sufficient for the characterization, since only two assays are required to pinpoint the difference between UFR and FR samples. Furthermore, standard statistical analysis is sufficient, and there is no need to apply more complicated ML methods.

In this study, the fermented and unfermented samples did not belong to the same origin of plant material and may contribute to the variation of TPC and AOC observed. Nonetheless, the data indicates major trends of TPC and AOC based on a large number of randomly selected samples. The obtained data also reflect the urgent need of finding well-defined procedures that reflect the actual values such as TPC and AOC of rooibos and other beverage teas in the market. These procedures should take into account the fluctuation of plant active constituents and different manufacturing processing and include simple and well-defined assays.

## Figures and Tables

**Table 1:** Factor correlation estimates for FR and UFR samples for different solvents.

Var 1	Var 2	FR/UFR	Solvent	*R* ^2^	$\sigma_{R^{2}}$	*p*-Value
TPC	TEAC	UFR	*H_2_O*	0.973	0.032	<1 × 10^−10^
TPC	FRAP	UFR	*H_2_O*	0.929	0.051	<1 × 10^−10^
TEAC	FRAP	UFR	*H_2_O*	0.922	0.054	<1 × 10^−10^
TPC	TEAC	FR	*H_2_O*	0.919	0.055	<1 × 10^−10^
TPC	FRAP	FR	*H_2_O*	0.934	0.050	<1 × 10^−10^
TEAC	FRAP	FR	*H_2_O*	0.928	0.052	<1 × 10^−10^
TPC	TEAC	UFR	*MeOH*	0.219	0.137	0.110
TPC	FRAP	UFR	*MeOH*	0.116	0.139	0.404
TEAC	FRAP	UFR	*MeOH*	0.708	0.099	<1 × 10^−10^
TPC	TEAC	FR	*MeOH*	0.691	0.101	<1 × 10^−10^
TPC	FRAP	FR	*MeOH*	0.212	0.139	0.122
TEAC	FRAP	FR	*MeOH*	−0.012	0.140	0.929

**Table 2:** Summary statistics for different assay values for the 47 UFR and 51 FR rooibos samples.

**UFR Water**				**UFR Methanol**		
	**TPC ^1^**	**TEAC ^2^**	**FRAP ^3^**	**TPC ^1^**	**TEAC ^2^**	**FRAP ^3^**
min	179.04	1672.29	338.90	208.46	1826.69	585.304
max	433.09	3549.76	949.89	414.29	2821.88	1473.89
median	274.07	2415.17	538.32	297.84	2396.97	885.732
average	282.08	2443.58	538.78	302.66	2421.41	899.17
**FR Water**				**FR Methanol**		
	**TPC ^1^**	**TEAC ^2^**	**FRAP ^3^**	**TPC ^1^**	**TEAC ^2^**	**FRAP ^3^**
min	174.04	1904.19	360.61	190.16	1210.58	460.310
max	558.41	5133.38	1306.71	300.242	2260.94	925.56
median	269.43	2834.59	631.67	259.481	2013.77	609.915
average	278.89	2871.18	647.50	256.815	1965.38	622.654

^1^ GAE/g; ^2^ TE/g; ^3^ AAE/g.

## Data Availability

The raw data presented in this study are available on request from the corresponding author.
